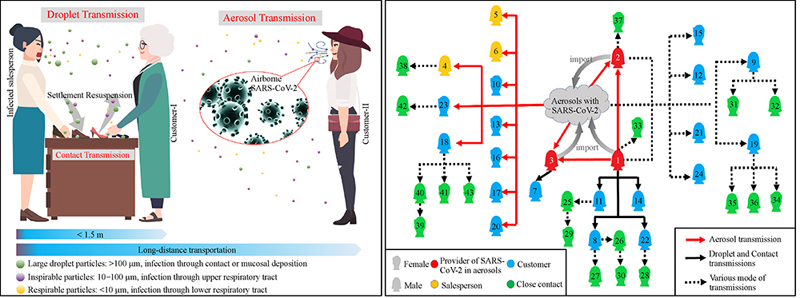# Aerosol transmission, an indispensable route of COVID-19 spread: case study of a department-store cluster

**DOI:** 10.1007/s11783-021-1386-6

**Published:** 2020-12-25

**Authors:** Guanyu Jiang, Can Wang, Lu Song, Xing Wang, Yangyang Zhou, Chunnan Fei, He Liu

**Affiliations:** 1 School of Environmental Science and Engineering, Tianjin University, Tianjin, 300350 China; 2 Tianjin Key Laboratory of Indoor Air Environmental Quality Control, Tianjin, 300350 China; 3 Tianjin Haihe Hospital, Tianjin, 300350 China; 4 Tianjin Centers for Disease Control and Prevention, Tianjin, 300350 China

**Keywords:** SARS-CoV-2, COVID-19, Environmental factor, Aerosol transmission, Epidemiologic characteristic

## Abstract

Patients with COVID-19 have revealed a massive outbreak around the world, leading to widespread concerns in global scope. Figuring out the transmission route of COVID-19 is necessary to control further spread. We analyzed the data of 43 patients in Baodi Department Store (China) to supplement the transmission route and epidemiological characteristics of COVID-19 in a cluster outbreak. Incubation median was estimated to endure 5.95 days (2–13 days). Almost 76.3% of patients sought medical attention immediately uponillness onset. The median period ofillness onsetto hospitalization and confirmation were 3.96 days (0–14) and 5.58 days (1–21), respectively. Patients with different cluster case could demonstrate unique epidemiological characteristics due to the particularity of outbreak sites. SRAS-CoV-2 can be released into the surrounding air through patient’s respiratory tract activities, and can exist for a long time for long-distance transportation. SRAS-CoV-2 RNA can be detected in aerosol in different sites, including isolation ward, general ward, outdoor, toilet, hallway, and crowded public area. Environmental factors influencing were analyzed and indicated that the SARS-CoV-2 transportation in aerosol was dependent on temperature, air humidity, ventilation rate and inactivating chemicals (ozone) content. As for the infection route of case numbers 2 to 6, 10, 13, 16, 17, 18, 20 and 23, we believe that aerosol transmission played a significant role in analyzing their exposure history and environmental conditions in Baodi Department Store. Aerosol transmission could occur in some cluster cases when the environmental factors are suitable, and it is an indispensable route of COVID-19 spread.

## 1 Introduction

Since December 2019, the fulminant epidemic of COVID-19 with the novel coronavirus (SARS-CoV-2) has been occurred at large scale. Then, the COVID-19 epidemic was rapidly spread the world (Bassetti, et al., 2020; Wang et al., 2020;Zhu et al., 2020). The World Health Organization (WHO) has announced that COVID-19 outbreak has become pandemic from March 11, 2020. Excitingly, the increasing population of confirmed patients with COVID-19 in China was well controlled after series of strict control measures. But on the contrary, patients with COVID-19

✉ Corresponding author
E-mail: wangcan@tju.edu.cn

Special Issue—Bioaerosol, Environment and Health (Responsible Editors: Can Wang, Jungho Hwang, Jingkun Jiang & Maosheng Yao)

were also increasing sharply in many other countries, leading to widespread concerns in global scope (Bhagavathula et al., 2020; Lau et al., 2020; Liu et al., 2020a; Phan et al., 2020; Rothe et al., 2020). The transmission modes and epidemiological characteristics of COVID-19 should be urgently explored to control its development.

Droplet and contact transmission are confirmed ways of COVID-19 transmission, whereas aerosol transmission as a potential route requires further confirmation (Jiang et al., 2020a). In addition, the relative importance of the mode of these transmissions is still unclear (Tellier et al., 2019). Case cluster exposure in hospitals, communities, department stores, and public transportation have continuously increased during the epidemic. These cases have counted a great number of available data. A cluster outbreak in Baodi Department Store in Tianjin affected more than 40 persons, including salespersons, customers, and their close contacts. However, a report that provides a satisfactory explanation of the feature of the epidemic outbreak in the area is not currently available. Thus, the epidemiological characteristics of COVID-19 in cluster case should be urgently explored, and its transmission routes in various cluster cases should be analyzed.

Herein, we provide an analysis of available data on the 43 confirmed patients of Baodi Department Store (China) cluster outbreak to clarify epidemiological characteristics and transmission routes of COVID-19 explored in this typical cluster outbreak.

## 2 Methods

### 2.1 Data collection

Tianjin Centers for Disease Control and Prevention (TCDC) and Tianjin Haihe Hospital (THH) were authoritative organizations for the prevention and control of COVID-19 epidemic in Tianjin, China. TCDC is in charge of epidemiological investigations of suspected and confirmed patients. A detailed epidemiological investigation will be carried out as soon as the suspected/confirmed patient was admitted to the THH.

Through interviews with patients, relatives, close contacts and health care workers, as well as the compilation of patient's medical records, we collected the information of patient's age, gender, occupation, clinical symptoms, onset date, first medical treatment date, first hospital visit date, hospitalization date and clinical results. Investigators interviewed patient's relatives as necessary to determine the contact history two weeks before the onset of epidemic. To detect the infection source of the patients in accurate ways as possible, we investigated the particular households and places where the patients went to visit for two weeks before outbreak onset and cross-analyzed the exposure history of all patients. Specially, in the initial traceability work, TCDC immediately retrieved the surveillance videos of Baodi Department Store from 20th–25th, January to determine whether the confirmed salespersons contact directly. We made further result analysis of results in this study based on the complete epidemiological investigation results and the preliminary clinical diagnosis data. To protect personal privacy, we omitted identity information and replaced it with numbers.

### 2.2 Study population

We carefully screened out 43 cases that related to Baodi Department Store out of 131 confirmed cases based on the results of the epidemiological investigation. We retrospectively analyzed the epidemiological characteristics of confirmed 43 patients with COVID-19 who were in direct and indirect contact with Baodi Department Store, typically including: 6 salespersons (case numbers 1 to 6), 18 customers (case numbers 7 to 24), and 19 of their close contacts (case numbers 25 to 43). The salespersons had sustained exposure in Baodi Department Store. The customers were only present in the specific area for a short-term, and their close contacts have realistically been second-generation infections without potential exposure to Baodi Department Store.

### 2.3 Epidemiologic analysis

We drew a Baodi Department Store plan with the accurate exposure site of salespersons and customers to describe the distribution of each functional zone and the infected area of patients in this store. We illustrated the ventilated conditions and the outbreak process of COVID-19 of Baodi Department Store. We described the characteristics, including demographic characteristics, exposures, and clinical diagnosis, of the infected patients onto a standardized table. We drew a diagram to expound the key date points relating to the epidemic identification of the patients during the timeline of illness. We drew a box-plot to analyze the key periods of these patients from infection to illness, including incubation, the onset to the first medical therapy, onset to hospitalization, and onset to confirmed date, confirmed utilizing the GRAPHPAD software. We combined surveillance video analysis and epidemiological investigation results to determine the infection route of 43 patients, and draw conclusions about transmission relation and route among patients.

## 3 Results

Functional areas were divided into nine parts, including ornament, costume, shoes, jewelry, clock, small appliance, cosmetic, headwear and luggage areas in Baodi Department Store. The adjacent areas were separated at least 1.5 m by a corridor, except for the costume and shoes area and the jewelry and clock area. No distinct boundary was present between the costume and shoes area and the jewelry and clock area (Fig. 1). Six salespersons were affected, and four of them (case numbers: 1, 4, 5, and 6) were working at the costume and shoes area, one (case number 3) was at the jewelry area, and one (case number 2) was assigned to the small appliance area. Eleven of the customers (case numbers: 7, 8, 10, 11, 13, 14, 17, 18, 19, 20 and 23) have been to the costume and shoes area. Two (cases numbers: 7 and 17) were to the jewelry area, and one (case number 7) was to the headwear area. In addition, we did not determine the accurate exposure areas of eight infected customers (case numbers: 9, 12, 15, 19, 21 and 24) (Fig. 1). The Baodi Department Store exposure date of salespersons and customers were marked as blue block and green circle, respectively.

The median ages were 50 years (range: 10–90) for the patients, 42 years (range: 34–52) for the salespersons, 50 years (range: 26–69) for the customers, and 51 years (range 10–90) for their close contacts. Among the 43 patients, 28 (65.1%) were female. Among the 20 customers, 16 (80%) were female, and all of the infected salespersons were women. The patients infected in this case were middle-aged and elderly, major to report exposure to the costume and shoes area, and major to be female. The clinical outcome was primarily slight and common types, whereas few cases revealed a severe type (Tables 1(a) and 1(b)).

Case number 1 was regarded as the imported source of infection in this cluster outbreak. Case number 1 contacted with a hyperpyrexia patient on business from January 12 to January 13, showed clinical symptoms on January 21st. Since all of the customers who had been to the store before January 20th had not been infected, we believed that SARS-CoV-2 began to spread in the environment of Baodi Department Store from January 20th. Therefore, case number 1 showed infection during the incubation period on January 20th (Fig. 2). Case numbers 2 and 3 were infected through long-term exposure to aerosol containing SARS-CoV-2 released by the case number 1. Then, they became the other major providers of airborne SARS-CoV-2 starting two days before they showed clinical symptoms. With the exception of case number 1, we explored the infection route of other 42 patients and divided them into 3 groups: group 1: most likely infected through aerosol transmission (case numbers: 2–6, 10, 13, 16, 17, 18, 20 and 23), group 2: most likely infected through droplet or contact transmission (case numbers: 7, 8, 11, 14 and 22), and group 3: infected through unclear transmission which means patients possibly infected by all routes (case numbers: 9, 12, 15, 19, 21, 24, and 25–43).

Given that all of the salespersons, were infected through prolonged exposure in Baodi Department Store (case numbers 2 to 6), except for case number 1. Case number 43 was an asymptomatic infected patient, and case number 33 did not have a clear source, we could not concretely determine their infection time and incubation period. Therefore, we only analyzed the incubation of all patients, except case numbers 2, 3, 4, 5, 6, 33, and 43.

The median of incubation was estimated to be 5.95 days (2–13; Fig. 3(a)). Almost 76.3% of the patients immediately sought for medical therapy as the outbreak onset, except for the patients who showed onset during an isolation period (case numbers: 33, 34, 35, 38, and 43; Fig. 3(b)). The median periods of illness onset to hospitalization and to confirmation were 3.96 (0–14) and 5.85 days (1–21), respectively (Figs. 3(c) and 3(d)). The result of this distribution period of customers, salespersons and their close contacts were similar across all of the patients without a significant difference.

**Fig. 1** Functional areas division and patients' exposure places in Baodi Department Store.
The distance between two adjacent areas is at least 1.5 m, except that there is no distinct boundary between the costume and shoes area, the jewelry and clock area. Case numbers 9, 12, 15, 19, 23, 24, and 26 did not provide their accurate exposure area.

**Table 1(a)** Characteristics of patients with COIVD-19 in Baodi Department Store

| Characteristic | Total ($N$ = 43) | Salespersons ($N$ = 6) | Customers ($N$ = 20) | Close contacts ($N$ = 17) |
|---|---|---|---|---|
| Median age (range) — yr | 50(10–90) | 42(34–52) | 50(26–69) | 51(10–90) |
| Age group — No./total No. (%) < 15 yr | 1/43(2.3) | 0/6(0) | 0/20(0) | 1/17(5.9) |
| Age group — No./total No. (%) 15–44 yr | 16/43(37.2) | 4/6(66.7) | 6/20(30) | 6/17(35.3) |
| Age group — No./total No. (%) 45–64 yr | 17/43(39.5) | 2/6(33.3) | 11/20(55) | 4/17(23.5) |
| Age group — No./total No. (%)⩾65 yr | 9/43(20.9) | 0/6(0) | 3/20(15) | 6/17(35.3) |
| Female sex — No./total No. (%) | 28/43(65.1) | 0/6(0) | 16/20(80) | 6/17(35.3) |
| Clinical outcome — No./total No. (%) Severe type | 3/43(7.0) | 2/6(33.3) | 0/20(0) | 1/17(5.9) |
| Clinical outcome — No./total No. (%) Common type | 20/43(46.5) | 2/6(33.3) | 10/20(50) | 8/17(47.1) |
| Clinical outcome — No./total No. (%) Slight type | 20/43(46.5) | 2/6(33.3) | 10/20(50) | 8/17(47.1) |

**Table 1(b)** Patients' exposure in Baodi Department Store

| Exposure to Baodi Department Store | Total ($N$ = 26) | Salespersons ($N$ = 6) | Customers ($N$ = 20) |
|---|---|---|---|
| Costume and shoes area— No./total No. (%) | 13/26(50) | 4/6(66.7) | 9/20(45) |
| Home appliance area — No./total No. (%) | 1/26(3.8) | 1/6(1.67) | 0/20(0) |
| Jewelry area — No./total No. (%) | 3/26(11.5) | 1/6(1.67) | 2/20(10) |
| Headwear area — No./total No. (%) | 1/26(3.8) | 0/6(0) | 1/20(5) |
| Unknown area — No./total No. (%) | 10/26(38.5) | 0/6(0) | 10/20(50) |

Notes: Reduced denominators indicate missing data; Percentages may not total 100 because of rounding.

Moreover, we have summarized some airborne SARS-CoV-2 detection results in multiple environment air samples, which proved that SARS-CoV-2 could be truly carried by air and cause the possibility of aerosol transmission.

## 4 Discussion

Although the previous retrospective studies have described the epidemiological, clinical, and transmission dynamics of COVID-19 patients in metropolitan areas, these characteristics have not also been analyzed in public cluster outbreak cases at the national level (Chan et al., 2020; Chen et al., 2020; Huang et al., 2020; Li et al., 2020; McMichael et al., 2020;Pan et al., 2020; Xu et al., 2020a). Here, we make the supplemental transmission route evaluation, dynamics and the epidemiological characteristics of COVID-19 in a cluster outbreak. What we found, it could supply a couple of significant points for further analysis in a cluster outbreak, including elucidate routes of transmission, control further spread, assess the effectiveness of control measures and predict the spread of infection in the future.

### 4.1 Epidemiological characteristics in this public cluster outbreak

Among the 43 COVID-19 patients in this cluster outbreak, we observed more females than males (Table 1). The result is slightly different from the earlier researches, where they found that more males were infected rather than females (Chen et al., 2020; Huang et al., 2020; Li et al., 2020; Xu et al., 2020a). Notably, the 22 out of all 26 infected salespersons and customers were female (84.6%), and half of them was in the costume area in Baodi Department Store. Out of the 17 close contacts of infected salespersons and customers, 11 (58.8%) were males. The cluster outbreak of COVID-19 that occurred in Baodi Department, a place where it showed more attractive to women. This was a satisfactory explanation for the abnormal gender proportion.

Most of the patients were classified in middle- aged and older people, but no older than 65 years (76.7%). The infected salespersons were generally young (Table 1). The clinical outcome was also consistent with other retrospective studies: a large proportion of the patients had slight and common symptoms (Table 1) (Chen et al., 2020; Huang et al., 2020; Li et al., 2020; Xu et al., 2020a). The two severe cases were identified, which both appeared in salespersons, indicating that long-term and stuffiness exposure may increase the risk of infection with severe symptoms.

Although the time interval in illness onset to first medicine therapy were generally short (81.4% of patients choosing to self-medicate within two days of symptoms), the time interval for hospitalization was long (average number of days was 4). Half of the patients were not hospitalized until at least 5 days of illness onset, and 23.3% of the patients were confirmed after detection in multiple

**Fig. 2** The key dates relating to the epidemic identification of COVID-19.
The infection time was consistent with exposure time in Baodi Department Store for customers, because all of them had the single dangerous exposure place before the first onset. The infection time of salespersons was not confirmed due to their long-term exposure in Baodi Department Store, except case number 1. Case number 43 was an asymptomatic infected patient and case number 33 did not have a clear source, we could not determine their infection time and incubation period in a conclusive way. A circle with two colors means the two events happening on the same day.

respiratory specimens (Fig. 3). Our finding suggests that in the early stages of illness, the identification and isolation of the disease cases are difficult. Nine-tenths of patients prefer to self-medicating rather than seeking professional medical care, which increases the difficulty of case screening, and transmission risk. Increasing the awareness on actively seeking medical attention is also important in the prevention and control of COVID-19.

**Fig. 3** Key periods distribution of epidemic development: (a) Incubation period; (b) Onset to the first medicine therapy; (c) Onset to hospitalization; (d) Onset to confirmation.

The incubation period was analyzed through all customers, close contacts and case number 1 in Panel A, because the incubation of salespersons was unknown, except case number 1. The onset to medicine therapy in patient is shown in Panel B, except case numbers: 33, 34, 35, 38 and 43 (they were onset during isolation period). The period of onset to hospitalization is shown in Panel C. The period of onset to confirmation is shown in Panel D.

### 4.2 Transmission routes of SARS-CoV-2 in cluster outbreak

COVID-19 has been defined as a respiratory infectious disease, and SARS-CoV-2 as a novel type of coronavirus is mainly released from a patient's body into the air through the respiratory tract, and it could load on exhaled droplets for more isolated transportation (familiar with SARS-CoV and MERS-CoV) (Rockx et al., 2020). Therefore, the droplets that emitted from the respiratory tract are used as the starting point for the transmission of infectious diseases, and exploring its characteristics in the air is of great significance for the analysis of disease transmission routes.

At present, there has been many studies clarifying the exhaled particle amount and size distribution of droplets that discharged through the respiratory tract (Roy and Milton, 2004; Fabian et al., 2008; Gralton et al., 2011; Gralton et al., 2013; Milton et al., 2013). It could be concluded as follows: 1) The size range of the droplets discharged through natural respiratory activities is less affected by health status, gender, age and sex. 2) Almost all of respiratory activities (breathing, coughing, sneezing and talking) can release droplets with a particle size ranging from < 0.1–100 μm, while it can be dispersed large droplet particles (>100 μm) into air in more violent coughing, sneezing and talking activities. 3) The number of particles produced by different respiratory activities is quite different: a cough can produce 3500 infectious particles, which is equivalent to 5 minutes of normal breath emissions, and a sneeze can emit 1 million particles into the surrounding air. 4) Some pathogenic microorganism can be emitted into the air via respiratory activities.

Respiratory tract infectious diseases may exist in multiple routes of transmission in many closed indoor environments, such as in ICU ward, steamship, department store and so on. In Baodi Department Store cluster outbreak, the main potential transmission routes will be droplet transmission, contact transmission, and aerosol transmission, if we do not take into account the use of the toilet places and concentrate on the shopping area. In droplet transmission, the particle size is larger, and the propagation range is smaller ( < 1.5 m) compared with that occurs in aerosol transmission (small particle size, and lager transportation range). But there is no specific boundary between the droplet transmission and aerosol transmission. According to the droplet size of 5 μm, WHO distinguishes the long-distance aerosol transmission

(≤5 μm) and close-range droplet transmission (>5 μm) (Siegel et al., 2007;World Health Organization., 2014), which is different with the 10 μm boundary defined by the Infectious Diseases Society of America (IDSA) on whether the particles can be deposited in the lower respiratory tract (Tellier et al., 2019). IDSA defines “respirable particles” as having a diameter of 10 μm or less and the diameter of “inspirable particles” is between 10 μm and 100 μm, while almost of “inspirable particles” are deposited in the upper respiratory tract.

Bioaerosol is a mixing decentralized system with aerodynamic diameter within 100 μm suspended in the air, which containing numbers of droplets, droplet nuclei and multiple inorganic compositions. Infectious particles no larger than 100 μm (such as respirable particles and inspirable particles), could be spread over long distances by bioaerosol. The susceptible population has infected by inhaling the pathogenic particles in the aerosol without closing contact with the infected person, thus completing the process of aerosol transmission. During the close contact between virus carriers and susceptible population, droplet transmission and contact transmission may happen: 1) The large droplet particles have a better settling performance and relative strong pathogenicity, which could be easily inhaled by the susceptible population or deposit on the eyes, mouth or nasal mucosa thereby causing the susceptible population being sick by droplet transmission. 2) The susceptible population may contain the virus in their own hands by directly (contacting like, shaking hands) or indirectly (touching virus droplets deposited on the object’s surface) ways, then, the virus travels through the hands and inoculates themselves into the mucous membranes (eyes, nose and mouth), causing an infection (Fig. 4).

### 4.3 Aerosol transmission in the cluster outbreak

Baodi Department Store is a 40-year-old commercial place located in the triangular region of Beijing, Tianjin, Tangshan. Baodi Department Store opened as usual before January 26th, 2020, and has been closed since January 26th, due to the COVID-19 epidemic outbreak (Fig. 2). Chinese Center for Disease Control and Prevention published a report indicating that infectivity in the incubation period of COVID-19 may occur in two days earlier before the first clinical onset of symptoms. This condition may seem to be the reason of the minimum time interval between the contact date and the onset date, endure three days in the

**Fig. 4** The transmission of exhaled virus between infected and susceptible population.
Infected salesperson could release the virus into the environment by violent expiration (cough, sneeze, talk) or simply exhalation. Customer-I are infected through close encounter (occurred droplet transmission or contact transmission). Customer-II are infected through sniffing airborne SARS-CoV-2 (aerosol transmission). The transportation distance of droplet particles usually less than 1.5 meters. Inspirable and respirable particles could spread through the aerosol for long distances, while droplet particles could transform into inspirable or respirable by evaporation. Deposited droplet particles could be re-suspended through human activity and air agitation.

infected customers (Fig. 2). The exposure of the 18 infected customers in Baodi Department Store was concentrated from January 20–25th, 2020, it was the period that the first three confirmed salespersons (case numbers: 1, 2 and 3) showed infectivity, and it was a time for purchasing goods for the people in preparation for the Spring Festival, suggesting a high population density (Fig. 2).

After analyzing the working dates and onset date of these salespersons, we found that three salespersons (case numbers: 4, 5 and 6) showed clinical symptoms after January 29th, which suggested they were not infectious during January 20th–25th. Consequently, the only half of them could meet the requirements as the source of infection (from January 20th–22nd for case number 1, January 20th–23rd for case number 2, and January 23rd–24th for case number 3) during the period of January 20th–24th. Therein, case number 1 was the first onset salesperson and possessed the capacity to infect susceptible from January 20$^{th}$, 2020. Case numbers 2 and 3 were working in diverse areas with the case number 1. Through field visits, case numbers 1, 2 and 3 did not retain any relationship by each other, and any close contact behaviors had not been recorded by the surveillance video (including, talking, eating at the same place, and using the common toilet). Therefore, the routes of the droplet and contact transmission can be essentially eliminated when speculated the infection routes of the case numbers 2 and 3.

Case number 1 released SARS-CoV-2 that living in his nasopharynx, respiratory tract and pulmonary to atmosphere by violent expiration (cough, sneeze, talk) or simply exhalation when infectivity was immediately shown. A part of SARS-CoV-2 which attached to nuclei particles in aerosols could travel considerable distance (over several meters) by aerosol transmission. Therefore, we believed that case numbers 2 and 3 were infected through long-term exposure to aerosol containing SARS-CoV-2. They were also the major providers of SARS-CoV-2 in aerosols after confirmed COVID-19 infection and showing infectivity.

Other patients in group 1 were similar with case numbers 2 and 3: any direct, in-plant, or indirect contact did not definitely occur. Nevertheless, patients in group 1 were infected through exposure to the aerosol and draw-in a number of SARS-CoV-2. Although the infectious characteristics of the incubation period of patients with COVID-19 were ambiguous, whether there was direct contact between salespersons and customers could be determined through exposure history analysis. Therefore, the transmission route of group 2 and 3 could be confirmed: there occurred in-plant or direct contact with an infection source in group 2; customers in group 3 did not provide their clear exposure area in the Baodi Department Store and close contacts may show various contact forms during their daily life (Fig. 5).

In summary, there is the evidence suggested that 12 out of 43 patients were most likely infected through aerosol transmission in this cluster outbreak. In the absence of indirect infection, the half of persons (12/24) were highly possible infected through aerosol transmission among the patients who were directly infected in Baodi Department Store. It suggested that aerosol transmission was an indispensable and important route for the proliferation of COVID-19.

Moreover, it is necessary to study the infection risk assessment of aerosol transmission for establishing prevention measures of COVID-19. Some previous reports have performed the theoretical analysis for the health risk assessment on aerosol transmission (Buonanno et al., 2020; Zhang et al., 2020). In our subsequent research, the estimation of the infection risk together with the model development of aerosol transmission will be performed.

### 4.4 Detected SARS-CoV-2 in aerosol

Many types of coronaviruses can survive and maintain their viral properties in the environment for 9 days (such as MERS-CoV and SARS-CoV) (Kampf et al., 2020). Nowadays, multiple research teams have detected nucleic acid positive for SARS-CoV-2 RNA in aerosol in different sites (Table 2).

Jiang et al. detected SARS-CoV-2 in the air where critically ill-patients were present (Jiang et al., 2020b). Guo et al. tested the air and surface swatches for the purpose of confirming the distribution of SARS-CoV-2 in hospital wards. They found that the intensive-care unit was more polluted than the normal ward (Guo et al., 2020). The airborne SARS-CoV-2 could not only be detected in indoor, but also in the outdoor environment. Setti et al. collected 34 of outdoor $PM_{10}$ swatches from an industrial base in Bergamo Province, and 5 of them presented the significant results of positivity after nucleic acid assay (Setti et al., 2020). They suggested outdoor air particles would be combined with airborne SARS-CoV-2 to form a polymer. This polymer had a low diffusion coefficient and propagation durability under stable atmospheric conditions. Santarpia et al. have recently clarified that the percent positive of air samples collected from the hallway and personal zone were 66.7% and 100%, respectively. And SARS-CoV-2 might be scattered in the environment in the form of expired particles when going to the toilet and in contact with pollutants (Santarpia et al., 2020). As for the granulometric distribution of SARS-CoV-2 in an indoor air environment, Chia et al. sampled from three airborne infection isolation rooms in the general ward, and SARS-CoV-2 positive particles with a particle size of $> 4$ μm and 1–4 μm were detected in 2 of 3 general wards with 12 times for ventilation per hour (Chia et al., 2020). It provided that SARS-CoV-2 could exist in the air and possess the capacity for long-term aerosol transmission.

**Fig. 5** The transmission chain in salespersons, customers and their close contacts
Case numbers 1, 2 and 3 are not only salespersons, but also providers of SARS-CoV-2 in aerosols. Group 1 consists of case numbers: 2–6, 10, 13, 16, 17, 18, 20 and 23; Group 2 is composed by case numbers: 7, 8, 11, 14 and 22; Group 3 is constituted by case numbers: 9, 12, 16, 15, 19, 21, 24 and 27 to 43. Various modes of transmissions between salespersons and customers mean that these customers exposure areas in Baodi Department Store were unclear, while aerosol, droplet or contact transmission are all possible

**Table 2** Positive SARS-CoV-2 RNA in aerosol at several sites

| Sites | Environmental description | Positivity/Total | Rate of positivity | References |
|---|---|---|---|---|
| Isolation ward | Patients in isolation underwent tracheal intubation the day before the samples | 1/20 | 5% | Jiang et al., 2020b |
| Intensive care unit | Indoor air near the air outlet | 5/14 | 35.7% | Guo et al., 2020 |
| | Indoor air near the patients | 8/18 | 44.4% | |
| | Indoor air near the doctors' office area | 1/8 | 12.5% | |
| General ward | Indoor air under the air inlet | 2/13 | 15.4% | |
| An industrial site of Bergamo Province | $PM_{10}$ samples | 5/34 | 14.7% | Setti et al., 2020 |
| Isolation ward | Isolation of asymptomatic or mildly ill | 80/107 | 74.8% | Santarpia et al., 2020 |
| Personal care unit | For individuals requiring hospital care | 34/40 | 85.0% | |
| Hallway Air Samples | Sampling at the same time as the ward sample collection | –/– | 66.7% | |
| Personal Air Samples | low volume personal air samples around patients | 7/7 | 100% | |
| Isolation ward | Sampling at three isolation wards; Samples from each room were pooled prior to analysis | 2/3 | 66.7% | Chia et al., 2020 |

## 4.5 Environmental factors impact on SARS-CoV-2 transmission

The virus survival time in the environment is affected by salinity, humidity, temperature, pH, solar radiation and air pollution (Sooryanarain and Elankumaran, 2015), while suitable environmental conditions such as worm, wet and stuffiness could lengthen the concentration and survival time of airborne SARS-CoV-2. Liu et al. detected that airborne SARS-CoV-2 RNA concentration around the patients' toilet, air was much higher than that in common wards with regular ventilation and in isolation units. They also found it was difficult to detect airborne SARS-CoV-2 RNA in most public places, except for two department stores which tend to get crowded (Liu et al., 2020b). Bukhari and Jameel found that 90% of the spread of COVID-19 within a certain temperature (3°C–17°C) and absolute humidity (4 g/m$^3$–9 g/m$^3$) (Bukhari and Jameel, 2020). They speculated that the high-level temperature and absolute humidity were to the disadvantage of SARS-CoV-2 transmission. Similarly, the finding of Ma et al. who showed the risk of SARS-CoV-2 was inversely proportional to the relative humidity and directly proportional to the diurnal temperature range (Ma et al., 2020). These studies have strongly demonstrated that the transmission of SARS-CoV-2 is influenced by ambient temperature and humidity.

Moreover, the weather conditions and ambient air pollution could also impact SARS-CoV-2 transmission. Xu et al. found that a U-shaped association among the estimated reproduction amount of COVID-19, outdoor UV contact, wind speed, sediments, diurnal changed temperature, $SO_2$ and ozone (Xu et al., 2020b). Yao et al. deliberated the influence of local environmental factors on the number of confirmed COVID-19 cases in 31 various regions of China, and the results suggested that the environmental diffusion coefficient of SARS-CoV-2 continued to decrease following the ambient ozone concentration increases (Yao et al., 2020).

In another research, the indoor virus concentration was affected by ventilation efficiency, and low-level ventilation rate would increase the danger of contact with potentially infectious droplet nuclei (Myatt et al., 2004). No air conditioning system which was installed in Baodi Department Store, indicating no good ventilation conditions. They had to keep the door closed and interdict the natural ventilation (thick windshield clothes were installed on the doors) to keep indoor rooms warm in the winter, which provided a suitable condition for the aerosol transmission of COVID-19. There also was no enough sunlight for indoor sterilization. No timely and regular sterilization has been conducted using disinfectants before the level 1 warning implemented on January 24th, in Tianjin.

However, the people were not aware or informed how to wear the mask during the period of this cluster outbreak, even though some types of masks such as N95, KF94, medical, surgical masks could limit the transportation of SARS-CoV-2 emitted by patient's respiratory activities (Leung et al., 2020). Therefore, SARS-CoV-2 discharged by patients breathing, talking, coughing, or sneezing was tolerant of living in aerosols in this suitable environment. These environmental factors provided excellent conditions for the aerosol transmission of COVID-19.

## 5 Conclusions

In summary, some epidemiological characteristics (such as symptoms, the incubation period and treatment time) are not significantly different among the epidemiological investigations in special cluster outbreak and large-scale areas. Based on the identification, the personal characteristics (such as age, sex and occupation) indicated its uniqueness due to the particularities of the outbreak site. Most of the infected customers are females, and the age range is relatively large, and the severe ill-patients are all salespersons in Baodi Department Store cluster outbreak. Droplet transmission, contact transmission, and aerosol transmission are main transmission routes in Baodi Department Store cluster outbreak, if we ignore the toilet places and focus on the shopping area. SRAS-CoV-2 can be released into the surrounding air through the patient's respiratory tract activities and can exist for a long time for long-distance transportation. SRAS-CoV-2 RNA can be detected in aerosol at several sites, such as in isolation ward, general ward, outdoor, toilet, hallway, and a crowded public area. The influence of environmental factors was analyzed and indicated that the SARS-CoV-2 transportation in aerosol was dependent on the temperature, air humidity, ventilation rate and inactivating chemicals (ozone) content. Indeed, the aerosol transmission is an important route, leading to the infection of a part of patients in Baodi Department Store cluster case.

**Acknowledgements** This research was supported by the Key Technologies R & D Program of Tianjin (No. 20ZXGBSY00100)

**Conflicts of Interest** All authors of this article have no conflicts of interest.

**Open Access** This article is licensed under a Creative Commons Attribution 4.0 International License, which permits use, sharing, adaptation, distribution and reproduction in any medium or format, as long as you give appropriate credit to the original author(s) and the source, provide a link to the Creative Commons licence, and indicate if changes were made. The images or other third party material in this article are included in the article's Creative Commons licence, unless indicated otherwise in a credit line to the material. If material is not included in the article's Creative Commons licence and your intended use is not permitted by statutory regulation or exceeds the permitted use, you will need to obtain permission directly from the copyright holder. To view a copy of this licence, visit http://creativecommons.org/licenses/by/4.0/.
